# Characterization of active joint count trajectories in juvenile idiopathic arthritis

**DOI:** 10.1186/1546-0096-10-S1-A44

**Published:** 2012-07-13

**Authors:** Roberta A Berard, George Tomlinson, Xiuying Li, Kiem G Oen, Alan M Rosenberg, Brian M Feldman, Rae SM Yeung, Claire Bombardier

**Affiliations:** 1 The Hospital for Sick Children, Toronto, ON, Canada; 2 London Health Sciences Centre, London, ON, Canada; 3 Royal University Hospital, Saskatoon, SK, Canada; 4 Toronto General Research Institute, Toronto, ON, Canada; 5 University of Manitoba, Winnipeg, MB, Canada

## Purpose

To describe the longitudinal active joint count (AJC) trajectories in juvenile idiopathic arthritis (JIA) and to examine the association of baseline characteristics with these trajectories.

## Methods

A retrospective cohort study at two Canadian centres was performed. The longitudinal trajectories of AJC were described using latent growth curve modeling (GCM). Latent GCM is a novel technique that aims to classify individuals into statistically distinct groups based on individual response patterns so that individuals within a group are more similar than individuals between groups. The trajectory classes are each defined by a longitudinal growth curve. The association of baseline characteristics stratified by trajectory group was examined by univariate methods.

## Results

Data were analyzed for 659 children diagnosed with JIA between 1990/03-2009/09. A maximum of 10 years of follow-up data were included in the analysis. Participants were classified into 5 statistically and clinically distinct AJC trajectories by latent GCM.

## Conclusion

Using a novel longitudinal statistical method we were able to classify patients with JIA based on their pattern of AJC over time. These results need to be interpreted in light of clinical significance. The trajectory classes will need to be examined for their predictive ability for distal outcomes and relationship to important genetic and biological predictors. Identification of patterns of disease course is important in working towards the development of a clinically relevant outcome-based classification system in JIA.

## Disclosure

Roberta A. Berard: None; George Tomlinson: None; Xiuying Li: None; Kiem G. Oen: None; Alan M. Rosenberg: None; Brian M. Feldman: None; Rae S.M. Yeung: None; Claire Bombardier: None.

